# AKT2 deficiency impairs formation of the BCR signalosome

**DOI:** 10.1186/s12964-020-00534-9

**Published:** 2020-04-06

**Authors:** Zuochen Du, Di Yang, Yongjie Zhang, Xingtian Xuan, Han Li, Leling Hu, Changshun Ruan, Liling Li, Anwei Chen, Liang Deng, Yan Chen, Jingwen Xie, Lisa S. Westerberg, Lu Huang, Chaohong Liu

**Affiliations:** 1 Chongqing Key Laboratory of Child Infection and Immunity, Children’s Hospital of Chongqing Medical University, Chongqing, China; 2 Department of Pediatric Research Institute, Children’s Hospital of Chongqing Medical University, Chongqing, China; 3 Ministry of Education Key Laboratory of Child Development and Disorder, Children’s Hospital of Chongqing Medical University, Chongqing, China; 4 International Science and Technology Cooperation base of Child development and Critical Disorders, Children’s Hospital of Chongqing Medical University, Chongqing, China; 5 Department of hematology and oncology, Children’s Hospital of Chongqing Medical University, Chongqing, China; 6 Department of Gastroenterology, The First Affiliated Hospital of Chongqing Medical University, Chongqing, China; 7 The Second Department of Pediatrics, Affiliated Hospital of Zunyi Medical University, Zunyi, GuiZhou Province China; 8 Clinical laboratory, Children’s Hospital of Chongqing Medical University, Chongqing, China; 9 Department of Microbiology Tumor and Cell Biology, Karolinska Institutet, Stockholm, Sweden; 10 Department of Pathogen Biology, Tongji Medical College, Huazhong University of Science and Technology, Wuhan, China

## Abstract

**Background:**

AKT2 is one of the key molecules that involves in the insulin-induced signaling and the development of cancer. In B cells, the function of AKT2 is unclear.

**Methods:**

In this study, we used AKT2 knockout mice model to study the role of AKT2 in BCR signaling and B cell differentiation.

**Results:**

AKT2 promotes the early activation of B cells by enhancing the BCR signaling and actin remodeling. B cells from AKT2 KO mice exhibited defective spreading and BCR clustering upon stimulation in vitro. Disruption of Btk-mediated signaling caused the impaired differentiation of germinal center B cells, and the serum levels of both sepecific IgM and IgG were decreased in the immunized AKT2 KO mice. In addition, the actin remodeling was affected due to the decreased level of the activation of WASP, the actin polymerization regulator, in AKT2 KO mice as well. As a crucial regulator of both BCR signaling and actin remodeling during early activation of B cells, the phosphorylation of CD19 was decreased in the AKT2 absent B cells, while the transcription level was normal.

**Conclusions:**

AKT2 involves in the humoral responses, and promotes the BCR signaling and actin remodeling to enhance the activation of B cells via regulating CD19 phosphorylation.

Video Abstract

## Background

AKT, also known as Protein kinase B (PKB), is an important kinase related to cell survival, growth and metabolism. The AKT family contains three highly conserved isoforms: AKT1 (PKBα), AKT2 (PKBβ) and AKT3 (PKBγ), all of them comprise three functional domains: a pleckstrin homology (PH) domain, a kinase domain-containing Thr308 (Thr309 in AKT2 and Thr305 in AKT3) and a C-terminal regulatory tail containing Ser473 (Ser474 in AKT2 and Ser472 in AKT3) [1]. AKT1 has a ubiquitous distribution, whereas AKT2 and AKT3 are expressed more limited. It has been found that AKT2 is highly abundant in embryonic brow fat, skeleton muscle and liver [2], and AKT3 is only highly expressed in brain and testis [3].

AKT2 is involved in type II diabetes and is one of the important kinases that transduce insulin-induced signals to regulate glucose and lipid metabolism [4, 5]. Patients with a loss-of-function mutation of AKT2 suffered from the autosomal dominant inheritance of severe insulin resistance and diabetes mellitus [6], and AKT2 KO mice have been reported to exhibit hyperglycemia, hyperinsulinemia and glucose intolerance [7]. Moreover, AKT2 is expressed highly in numerous types of tumor cells, and there is a strong correlation between the high expression of AKT2 and the development of the malignant tumors in the liver, pancreas and colon [8–10].

AKT2 plays an important role in the immune system. In the Salmonella acute gastroenteritis mice model, AKT2 KO mice are much more sensitive to the infection and exhibit high morbidity and mortality. This is associated with the recruitment of neutrophils and macrophages in the intestine after Salmonella infection [11]. The effects of AKT2 on the migration in both neutrophil and macrophage are well demonstrated using AKT2 knockout mouse models [12, 13]. Macrophages from mice devoid of AKT2 preferentially differentiate into the anti-inflammatory M2 subtype using the Ldlr^−/−^ mouse model, leading to slower progress of atherosclerosis [14]. However, the effect of AKT2 in B cells is poorly understood.

B cells are critical drivers of the humoral immunity by the generation of antigen-specific antibodies. Upon stimulation with antigen, B cell receptors (BCR) s on the cell surface are organized into membrane-tethered clusters by cross-linking, and the clustering induces the interaction of BCR with lipid raft and lipid raft-resident kinases which phosphorylates the tyrosine-based activation motif (ITAM) on BCR [15]. The activated BCRs recruit signal molecules to the membrane, and the key kinase Syk was activated when binding to the phosphorylated ITAM. Then, the downstream signal molecules recruited to BCR, such as PI3K, PLCγ2, Bruton’s tyrosine kinase (Btk) and CD19, are phosphorylated by the activated Syk [16]. The recruitment and activation of these signal molecules lead to form the BCR signalosome at the membrane and transduce the BCR signaling into the cytoplasm [17]. As BCR clusters aggregating with each other and move centripetally, a central cluster is finally formed in the pole of the cell [18]. Moreover, BCR signaling is tightly controlled by some inhibitory phosphatases, including phosphatidylinositol-5 phosphatase (SHIP), which is activated by the colligation of FcγRIIB [19]. The negative regulation of BCR signaling is critical to control the autoimmunity mediated by B cell [20].

Furthermore, many studies have reported that actin remodeling is involved in the initiation of BCR activation [21]. The cortical actin network, which supports the structure of the plasma membrane of B cells, physically influences the BCR lateral movement by creating temporary mobility barriers with actin-binding proteins in the resting B cells [22]. The binding of BCR with antigen promotes the BCR self-clustering by inducing the disassembly of actin network, followed by actin dramatic reassembly in the vicinity of newly formed BCR microcluster, especially at the outer edge of the B-cell contact zone [23]. The reassembly of actin leads to the spreading of B cells and provides opportunities for BCRs to bind with more membrane antigens. As BCR microclusters merge with each other, actin decreases around BCR clusters and reorganized around the outer edge of the BCR central cluster, which results in the centripetal movement of BCR clusters and contraction of B cells [24].

The dynamic actin remodeling during the early activation of B cells is regulated by several regulators. Wiskott-Aldrich syndrome protein (WASP), uniquely expressed in hematopoietic cells, is demonstrated to play important roles in the actin remodeling of B cells. As an actin regulator, the conformational changed WASP promotes actin polymerization by activating the Arp2/3 complex [25]. And reciprocal regulation was found between the dynamic actin remodeling and BCR signaling in the initiation of B cell activation as well. The key signal molecules Btk and SHIP-1 are proved to be essential for controlling actin remodeling upon BCR stimulation [24]. As the major stimulatory BCR co-regulator, CD19 is demonstrated involved in the early activation of B cells. The deficiency of CD19 leads to the defect of BCR signaling after cytoskeleton disruption [26], moreover, CD19 could mediate actin remodeling by activating WASP via cross-linking with BCR [27].

A previous study shows that AKT1 and AKT2 are needed for the generation of MZ B cells and B1 B cells [28]. Constitutive AKT1 signaling impairs B cell receptor signaling, proliferation and promotes B cell effector function [29]. AKT2 is demonstrated to be the main adaptor of mTORC2 signaling during B cell development in bone marrow [30], but the function of AKT2 in B cells have not been clearly described. In this study, we have investigated the function of AKT2 in B cells in AKT2 KO mice. The early activation of B cells was examined by internal reflection fluorescence microscopy (TIRFm). AKT2 deficiency led to an intrinsic decreased number of germinal center B cells and impaired early activation of B cells. We found that AKT2-deficient B cells showed reduced early BCR signaling with decreased spreading and actin polymerization, and the phosphorylation of CD19 and WASP were reduced in the AKT2-deficient B cells as well. Furthermore, the immunized AKT2 KO mice presented reduced serum content of both specific IgM and IgG. Our results indicated the important role of AKT2 in B cell early activation and humoral immune response, and suggest that AKT2 regulates Btk-mediated BCR signaling through CD19.

## Material and methods

### Ethics statement

All animal work was reviewed and approved by the Institutional Animal Care and Usage Committee of Children’s Hospital of Chongqing Medical University. It is in line with the principles of medical ethics and the various requirements of Helsinki Declaration.

### Mice

AKT2 knock-out mice on C57BL/6 background were purchased from Jackson laboratory. C57BL/6 wild-type mice were used as control, and AKT2 KO and control mice were bred and maintained in the animal facility in the Children’s Hospital of Chongqing Medical University.

### Bone marrow chimera mice

Generation of bone marrow chimera mice has been described previously [31]. Briefly, CD45.1 mice were irradiated with sublethal radiation (6Gy) and then intravenously injected with 1 × 10^7^ total BM cells containing AKT2 deficient BM (expressing CD45.2) and WT BM (expressing CD45.1) at a ratio of 1:1. The recipient mice were analyzed by flow cytometry after 8 weeks.

### Immunization and ELISA

Immunization and ELISA analysis were performed as described previously [32]. Briefly, WT and AKT2 KO mice at the age of 6–8 weeks were injected with 400 μg NP-KLH (Biosearch Technologies) in 400 μl Ribi Adjuvant (MPL + TDM Adjuvant System; Sigma) subcutaneously at day 1, after 14 days mice were accepted the same second immunization. Serum was collected on the fifth day after the secondary immunization, and ELISA was used to analyze the content of IgM and IgG1 with NP-bovine serum albumin–coated plates and Ig isotype-specific secondary Ab (Southern Biotech).

### Phospho-flow cytometry

Splenic B cells were isolated as previously described [33]. Fresh splenic B cells were incubated with biotinylated F (ab’)_2_ fragment goat anti-mouse IgM and IgG (mBiotin-F (ab’)_2_-anti-Ig), followed by incubation with or without streptavidin. After activation for varying length of times, cells were fixed, permeabilized and blocked with Fcγ receptor (FcγR) antibodies (anti-mouse CD16/CD32; BD Bioscience), and then incubated with anti-pY (Millopore), anti-pCD19 (Abcam) or anti-pBtk (Abcam). The cells were measured and analyzed by flow cytometry.

### Total internal reflection fluorescence microscopy

Images were obtained as previously described [32].

### Confocal

Splenic B cells were isolated as previously described [33]. The purified B cells were seeded on the slides coated with poly-lysine, and incubated with biotinylated F (ab’)_2_ fragment goat anti-mouse IgM and IgG, labeled with Alexa Fluor 546 (AF546-mB-F (ab’)_2_-anti-Ig) and streptavidin. After activated, cells were fixed, permeabilized, and incubated with anti-pY (Millopore), anti-pCD19 (Abcam) or anti-pBtk (Abcam) antibodies. The image was obtained by CFm (Nikon).

### Real-time PCR

To make a comparison of the gene expression of *cd19, btk* and *was* in splenic B cells from WT and AKT2 KO mice, total RNA was isolated by RNAPURE kit (RP1202; BioTeke) from 1 × 10^6^ B cells, and was then reversed into cDNA by using PrimeScript™ RT reagent Kit (Takara). The gene expression was detected by CFX96 TouchTM equipment (Bio-Rad) with RT-PCR Assay Kit (Qiagen). *Gapdh* was used to standardize mRNA expression. Primers for *C*d19, *Btk* and *Was* were as follows: *Cd19* Forward 5′-GGACAGTGAACGTGGAGGAT-3′, *Cd19* Reverse 5′-GGGCACATACAGGCTTTGTT-3′. *Btk* Forward 5′-CGCCATTACGTTGTGTGTTC-3′, *Btk* Reverse 5′-TAGAAGGCGCGTTTTTGTTT-3′. *Was* Forward 5′-CCAACTGATAAGAAACGCTCAG-3′, *Was* Reverse 5′-CTGACATGTTTGAATCCACTCG-3′. *Gapdh* Forward 5′-TGCCCCCATGTTTGTGATG-3′, *Gapdh* Reverse 5′-TGTGGTCATGAGCCCTTCC-3′.

### Statistical analysis

Statistical significance was assessed by two-tailed unpaired Student’s t-test with Prism software (GraphPad Software, San Diego, CA), The *p* values were determined in comparison to WT (∗*p* < 0.05; ∗∗*p* < 0.01; ∗∗∗*p* < 0.001).

## Results

### AKT2 deficiency leads to decreased number of germinal center (GC) B cells and reduced serum levels of specific antibodies in mice

**Fig. 1:**
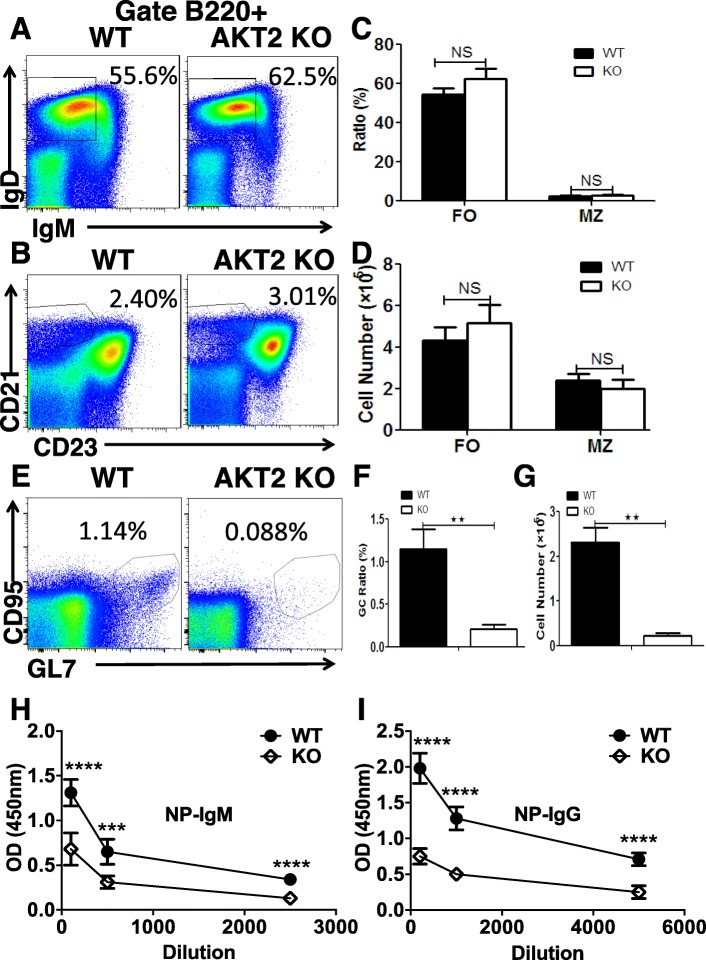
AKT2 deficiency leads to decreased number of germinal center (GC) B cells and reduced serum levels of specific antibodies in mice. The follicular (FO), marginal zone (MZ) and germinal center (GC) populations of B cells in spleen were detected by flow cytometry. Spleen cells from 8 week-old wild-type (WT) and AKT2 KO (AKT2-/-) mice (*n* = 6) were stained with B220, IgD, IgM, CD21, CD23, CD95 and GL7 antibodies (**a**, **b**, **e**). The percentage (**c**) and cell number (**d**) of FO B cells (IgD^+^ IgM^low^) and MZ B cells (CD21^+^ CD23^low^) in AKT2 KO mice were analyzed. GC B cells were marked as CD95^+^GL7^+^, and the percentage (**f**) and cell number (**g**) of GCB cells was reduced in AKT2 KO mice. Mean results from ELISA detecting NP-specific immunoglobulin M (IgM)(**h**) and Immunoglobulin G1 (IgG1) (**i**) from immunized WT and AKT2 KO mice (*n* = 6). The experiments were performed more than three times independently, and data are presented as mean ± SEM, **p < 0.01

To determine the effect of AKT2 on the differentiation of B cells in the periphery, we investigated the percentage and cell numbers of follicular B (FO) cells, marginal zone B (MZ) cells and germinal center (GC) B cells in the spleen from 8-week old wild-type (WT) and AKT2 KO mice. We found that AKT2 did not have any impact on the differentiation of FO and MZ B cells in mice (Fig. 1a-d), whereas non-immunized AKT2 KO mice had markedly reduced numbers and percentage of GC B cells (Fig. 1e-g). To determine whether the impact of GC B cells in AKT2 KO mice is cell-intrinsic or not, we generated WT-WT and WT-AKT2 deficient bone marrow CD45.1-CD45.2 mixed chimera mice, CD45.1 mice were used as the recipients. After 8 weeks of the transplantation, the mice were euthanized for flow cytometry analysis. The result showed there were no differences of the FO and MZ B cells in CD45.2 WT and AKT2 KO mice (Fig. S1A-D), but the percentage of GC B cells in CD45.2 AKT2 KO mice was decreased (Fig.S1E-F), which indicates that the impact of AKT2 deficiency on GC B cells is cell-intrinsic.

In order to further confirm the influence of AKT2 deficiency on humoral immune responses, we detected the serum level of NP-specific subclasses of the mice that were immunized with T-cell dependent antigen-4-hydroxy-3-nitrophenylacetyl-keyhole limpet hemocyanin (NP-KLH). Mice were immunized twice in 19 days, and the serum was collected and analyzed for the content of NP-specific subclasses at day 5 after secondary immunization. We found the levels of both NP-specific IgM and IgG were decreased in AKT2 KO mice (Fig. 1h-i). Taken together, these results indicate AKT2 deficiency affects the humoral immunity of mice.

### AKT2 deficiency impairs B cell spreading, BCR cluster formation and BCR signalosome during B cell early activation

**Fig. 2:**
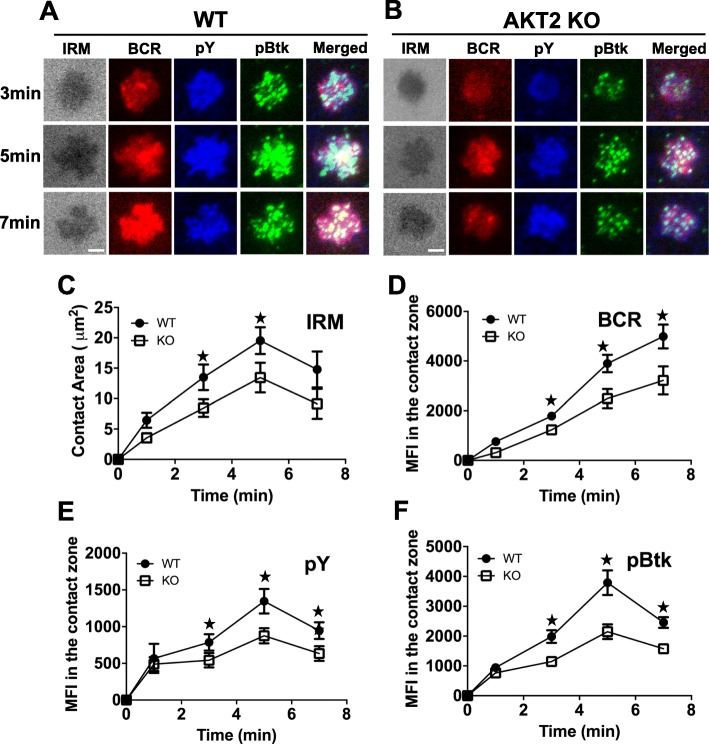
AKT2 deficiency impairs B cell spreading, BCR cluster formation and BCR signalosome during B cell early activation. Spleen cells from 8-week old WT and AKT2 KO mice (*n* = 3) were incubated with biotinylated Fab fragment goat anti-mouse IgM and IgG, labeled with Alexa Fluor 546 (AF546-mB-Fab’-anti-Ig) tethered to lipid bilayers. After incubation at 37 °C for varying lengths of time, cells were fixed, permeabilized, and stained with anti-pY and anti-pBtk antibodies. Shown are the respective images by TIRFm (**a, b**), and the B-cell contact zone area (**c**), mean fluorescence intensity (MFI) of BCR (**d**), pY (**e**) and pBtk (**f**) in the contact zone area were analyzed using IRM and TIRFm. Data were generated from > 50 cells from 3 individual experiments, and presented as mean values ± SEM, Scale bars, 2.5 μm. *p < 0.05

To determine the role of AKT2 in early BCR signaling, WT and AKT2-deficient B cells were stimulated with mAg and detected by total internal reflection fluorescence microscopy (TIRFm) (Fig. 2a-b). The contact area was measured by interference reflection microscopy (IRM). WT B cells spread rapidly after stimulation, and the contact area peaked at 5 min and then decreased as the cells contracted. On the contrary, B cells from AKT2 KO mice presented a reduced contact area at any time point, even though there was still a peak at 5 min after stimulation (Fig. 2c). Mean fluorescence intensity was used to show the BCR clustering and BCR signaling in the contact area upon stimulation. When compared to WT B cells, the BCR clustering in the contact area was reduced in the B cells from the AKT2 KO mice (Fig. 2d). By using antibodies for phosphotyrosine (pY) and phospho-Btk (pBtk), one of the important upstream signaling molecules in the BCR signaling pathway, we examined phosphorylation in the contact area upon stimulation. The MFI of pY and pBtk increased rapidly in the WT cells after stimulation with a peak at 5 min, while the MFI of pY and pBtk in the contact area were reduced in AKT2-deficient B cells, corresponding to decreased contact area (Fig. 2e-f). Meanwhile, by determining the mRNA expression of *Btk* in WT and AKT2-deficient splenic B cells, we found the gene expression of *Btk* was comparable between WT and AKT2 KO mice (Fig. S2A), indicating that AKT2 deficiency only affects the activation of Btk in B cells. These results reveal impaired early activation of B cells upon BCR stimulation of AKT2-deficient B cells from mice.

### BCR signaling is decreased in AKT2-deficient B cells

**Fig. 3:**
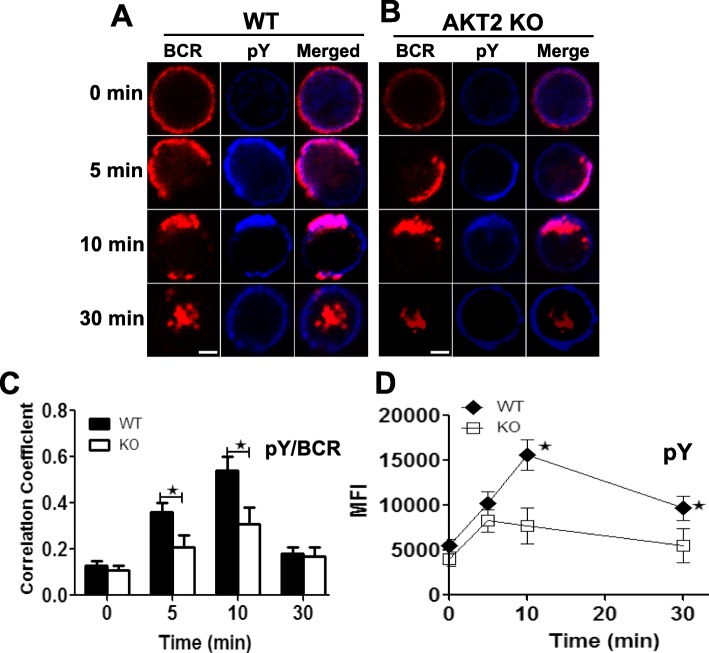
BCR signaling is decreased in AKT2 KO mice. The BCR signal molecular is detected by CFm (**a, b**) and flow cytometry (**d**). Spleen cells from WT and AKT2 KO mice (*n* = 3) were loaded on lysine-coated slides and incubated with AF546-mB-F (ab)_2_′-anti-Ig without (−) or with streptavidin, and then stimulated in 37 °C for indicated times. After fixed and permeabilized, cells were stained with anti-pY antibody and analyzed with CFm. The images of CFm are shown (**a, b**), and the Pearson’s correlation coefficients between BCR and pY in stimulated cells were determined by NIS-Elements AR 3.2 software (**c**). Data were generated from more than 50 cells from 3 independent experiments. Scale bars, 2.5 μm. Flow cytometry analysis of the MFI of pY from stimulated cells at different time points (**d**), and the average value (± SEM) was presented from 3 individual experiments, *p < 0.05

To further determine the BCR signaling in the AKT2 defective B cells, we used CFm to investigate the overall level of BCR signal activated by sAg. F (ab´)_2_-anti-Ig labeled with both mBiotin and Alexa Fluor 546 (AF546-mB-F (ab’)_2_-anti-Ig) was used as the sAg in this system, and the B cells incubated with the sAg would be activated by BCR cross-linking when incubated with streptavidin at the same time [34]. B cells from WT or AKT2 KO mice were stimulated for varying lengths of time within 30 min. We found that BCRs on the WT B cells surface clustered upon stimulation, and assembled on the cells surface at around 10 min, followed by internalization after 30 min. During the same time, the level of pY induced by BCR activation increased during the first 10 min in WT B cells. In contrast, we found that the pY fluorescence was much reduced in the AKT2-deficient B cells (Fig. 3a-b). Notably, the Pearson’s correlation coefficient between BCR and pY was reduced in the AKT2-deficient B cells (Fig. 3c). The MFI of pY in B cells stimulated with sAg was detected by flow cytometry. The level of pY had a peak at 10 min during activation in the WT B cells, however, it was significantly reduced in B cells in the absence of AKT2(Fig. 3d). Together, these results reveal that the BCR signal is reduced in the AKT2-deficient B cells.

### AKT2 deficiency leads to the defect of actin polymerization during BCR activation

**Fig. 4:**
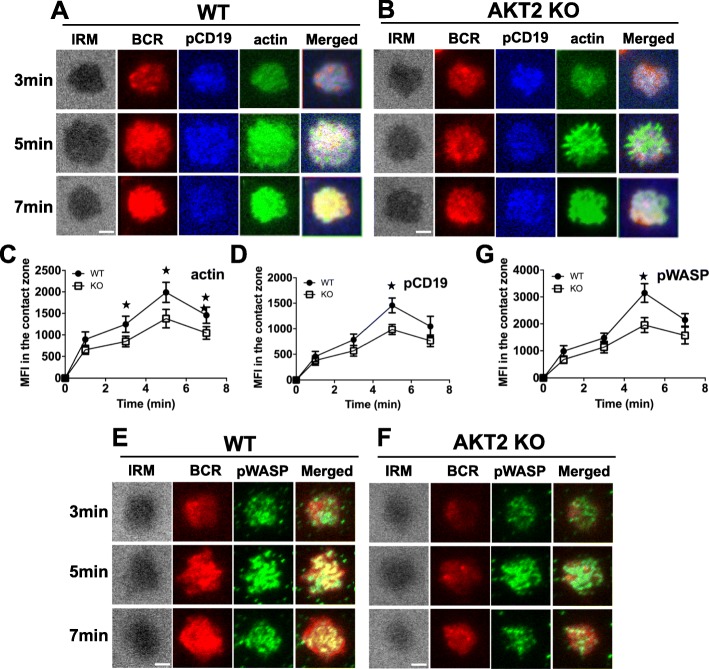
AKT2 deficiency leads to the defect of actin polymerization during BCR activation. TIRFm is used to detect the actin polymerization, the recruitment of phosphorated CD19 (pCD19) and phosphorated WASP (pWASP) in the contact zone of B cells during early B cell activation. Spleen cells from WT and AKT2 KO mice (*n* = 3) were incubated with AF546-mB-Fab’-anti-Ig tethered to lipid bilayers, following stimulated at 37 °C for varying length of times. After fixed and permeabilized, cells were stained with AF488-phalloidin for actin, anti-pCD19 and pWASP antibodies. Representative images of TIRFm were shown (**a, b**, **e, f**). The MFI of pCD19, actin and pWASP were analyzed by TRIFm (**c, d**, **g**), and data are generated from more than 50 cells from 3 individual experiments and presented as average value ± SEM, Scale bars, 2.5 μm. *p < 0.05

Actin reorganization is associated with the cell spreading and BCR clustering in the B cell early activation. Since the spreading of B cells and BCR clustering were impaired in the AKT2-deficient B cells, we next examined the F-actin content in B cells activated with mAg. The MFI of F-actin in the contact area of activated B cells was detected and analyzed using TIRFm (Fig. 4a-b). We found that actin polymerization increased within 5 min in both WT and AKT2-deficient B cells upon stimulation, and then decreased. In comparison with control cells, the MFI of F-actin was reduced at all time points in AKT2-deficient B cells (Fig. 4c).

WASP is an important regulator of the actin cytoskeleton via Arp2/3. To further investigate the effect of AKT2 on the actin cytoskeleton, we measured phosphorylated WASP (pWASP) in B cells activated by mAg. The MFI of pWASP in the contact area was significantly reduced in the AKT2-deficient B cells during activation (Fig. 4e-g). However, the gene expression of *Was* was not affected in AKT2-deficient B cells (Fig. S2B). Altogether, these results suggest that AKT2 influences actin polymerization during early B cell activation. Furthermore, the effect of AKT2 on actin polymerization in B cells may be mediated by phosphorylation of WASP.

### The phosphorylation of CD19 is decreased during early B cell activation in AKT2 KO mice

**Fig. 5:**
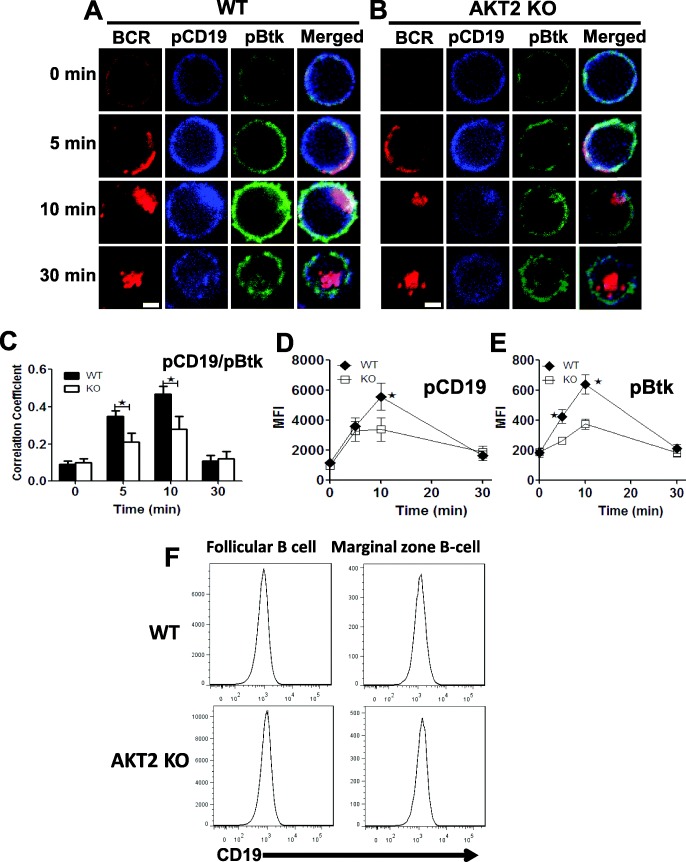
The phosphorylation of CD19 is decreased during B cell early activation in AKT2 KO mice. The phosphorylation of CD19 and Btk during B cell activation were determined by CFm and flow cytometry. **a** Spleen cells from WT and AKT2 KO mice (*n* = 3) were seed on the lysine coated slides, and incubated with AF546-mB-F (ab)_2_′-anti-Ig and streptavidin on ice. Cells were stimulated at 37 °C for indicated times, and then fixed and permeabilized, following stained with monoclonal antibodies of pCD19 and pBtk. Shown are the representative images of CFm (**a, b**). The Pearson’s correlation coefficients of pBtk and pCD19 at different time points during activation were analyzed by NIS-Elements AR 3.2 software (**c**). Data were generated from more than 50 cells from 3 independent experiments. Scale bars, 2.5 μm. Flow cytometry analysis of the MFI of pCD19 and pBtk of B cells stimulated by sAg (**d, e**). CD19 expression of FO B cells (B220^+^ IgD^+^ IgM^low^) and MZ B cells (B220^+^ CD21^+^ CD23^low^) was detected by flow cytometry (**f**). 3 independent experiments were performed, and data were presented as mean ± SEM, *p < 0.05

CD19 promotes the formation of BCR clustering, which further induces the BCR signaling. CD19 regulates BCR crosslinking, pWASP, and actin polymerization during the activation of B cells. Since both the BCR signaling and actin polymerization were affected in AKT2-deficient B cells, we further investigated the level of phospho-CD19 (pCD19) in B cells upon stimulation with sAg to determine whether AKT2 has a role in the activation of CD19. Using CFm, we found that the activation of both pCD19 and pBtk were reduced in AKT2-deficient B cells during 30 min of activation (Fig. 5a-b). The Pearson’s correlation coefficients between pCD19 and the activation of pBtk was analyzed with CFm, and the result showed the correlation between pCD19 and pBtk kept increasing within 10 min in WT B cells, and then decreased at 30 min. Although the correlation between pCD19 and pBtk increased in the first 10 min upon stimulation in AKT2-deficient B cells, the value of the correlation coefficients was reduced at 5- and 10-min time point after stimulation when compared to WT cells (Fig. 5c). The level of pCD19 and pBtk were further detected by flow cytometry, the MFI of pCD19 was only decreased at the 10 min time point (Fig. 5d), while the MFI of pBtk in AKT2-deficient B cells was significantly reduced at 5- and 10-min time points. (Fig. 5e). We next detected the mRNA expression of *Cd19* in WT and AKT2-deficient splenic B cells, and found no difference (Fig. S2C). Furthermore, the expression of CD19 in both follicular and marginal zone B cells respectively was examined using flow cytometry, notably, there was not any change of the expression of CD19 in AKT2-deficient B cells (Fig. 5f). Together, AKT2 might affect the activation of CD19 during B cell activation.

## Discussion

AKT2 has been well studied in cancer and insulin-mediated signaling, and in our study we demonstrate the function of AKT2 in B lymphocytes. The number of GC B cells was dramatically reduced in the ATK2 KO mice without immunization, whereas differentiation of marginal zone and follicular B cells was similar to wildtype mice. Using TIRFm, we provide evidence for that AKT2 deficiency in B cells led to the defective cell spreading, BCR clustering and signalosome formation after stimulation in vitro, and the BCR signaling and actin polymerization were reduced in the early activation. By generating AKT2 KO chimera mice, we found that the number of GC B cells was reduced in AKT2 KO chimera mice, meanwhile, the serum levels of NP-specific IgM and IgG were both reduced in the immunized AKT2 KO mice. Furthermore, we found that the phosphorylation of WASP, a critical mediator involved in actin polymerization, was reduced in the B cells absent of AKT2. Although the level of CD19 was not affected, the diminished level of phosphorylation of CD19 upon BCR signaling was evident. The present study indicates a positive effect of AKT2 on B cell responses, and provides a mechanism by which AKT2 mediates the early activation of B cells.

One report shows there is no effect of AKT2 on the development of early B cells in mice [28], and we provide evidence that AKT2 influenced the GC B cell differentiation in the periphery, and the effect is found to be cell intrinsic. Germinal center is the crucial space for the proliferation and differentiation of the antigen-specific B cells, and is essential for the humoral immune response. The reduction of GC B cells in AKT2 knockout mice indicates a defective humoral immune response. Therefore, we further detected the serum content of NP-specific subclasses of antibodies in the AKT2 KO mice immunized with T cell dependent antigen NP-KLH. The significant reduced levels of NP-specific IgM and IgG in the AKT2 KO mice indicates AKT2 participates in regulating the impaired humoral immune.

The early activation of B cells is initiated by BCR signaling and actin remodeling that regulates each other during activation [35]. In our study, AKT2 deficiency reduced both the BCR signaling and actin remodeling. AKT2 participates in regulating actin remodeling in neutrophils and macrophages, and AKT2 deficiency results in defective migration and chemotaxis [36, 37]. AKT2 influenced actin polymerization in B cells upon activation and this was associated with decreased B cell spreading. Without spreading, BCRs on the activated B cells would be in contact with fewer antigens, which would lead to the decreased BCR signaling. WASP is the downstream of BCR signaling and regulates actin polymerization [25]. Previous studies demonstrate that WASP-deficiency leads to defective B-cell migration, homing and humoral response [38], and we have previously shown that WASP mediates the early activation of B cells [39]. In the AKT2-deficient B cells, we found the phosphorylation of WASP induced by BCR was decreased.

CD19 is a co-receptor of BCR expressed on the B cell surface. The loss of CD19 leads to severe defects of humoral response in mice and humans. Not only the numbers of naive or the memory B cells are reduced in CD19-deficient mice, but the antigen response and antibody-producing capacity of B cells are also impaired in both mice and patients devoid of CD19 [40, 41]. Earlier study indicates that CD19 is essential for the BCR signaling induction by promoting the aggregation of BCR in the early activation [26]. Moreover, CD19 mediates the actin remodeling by regulating the activation of WASP through enhanced BCR crosslinking [27, 33]. Here we show that there is no change of the transcriptional expression of *CD19*, but the reduced phosphorylation of CD19 found in activated AKT2-deficient B cells provides a possible mechanism of the regulation. Nevertheless, AKT2 was reported as the downstream target of CD19-PI3K in BCR signaling [42]. Therefore, based on the data presented here, we suggest that there is positive feedback between AKT2 and CD19 in BCR-induced signaling, but the mechanism of the feedback loop needs to be further studied.

## Conclusions

In summary, our findings indicate the important role of AKT2 in the humoral immune response, and suggest that AKT2 regulates the early activation of B cells by regulating the BCR signaling and actin remodeling via CD19. Since AKT2 are involves in metabolic processes, further work is needed to understand the relationship between early activation of B cells, the cellular metabolism, and the effect of AKT2 on the humoral response.

## Supplementary information

**Additional file 1: Fig. S1.** The effect of AKT2 deficiency on the germinal center (GC) B cells is cell-intrinsic. CD45.1 mice were irradiated with sublethal radiation (6Gy), and then intravenously injected with 1 × 10^7^ total BM cells containing AKT2 deficient BM (CD45.2) and WT BM (CD45.1) at a ratio of 1:1. After 8 weeks, B cells from the chimera mice were labeled with specific antibodies for surface marker of FO (A), MZ (C) and GC (E) B cells, and analyzed using flow cytometry (*n* = 3). Shown are the frequency (+SEM) of different populations (B, D, F). T-test was used to do the statistics, *p < 0.01. **Fig. S2.** The gene expression of *Btk*, *Was* and *Cd19* is comparable in splenic B cells from WT and AKT2 KO mice. The total RNA was isolated from the splenic B cells from WT and AKT KO mice (n = 3). The gene expression of *Btk* (A), *Was* (B) and *Cd19* (C) were detected by real-time PCR, and *Gapdh* was used to standardized the mRNA expression. T-test was used to do the statistics.

## Data Availability

All data generated or analyzed during this study are included in this article.

## Abbreviations

AKT2: AKT serine/theonine kinase 2
BCR: B-cell receptor
BM: Bone marrow
Btk: Bruton tyrosine kinase
FO: Follicular
GC: Germinal center
WT: Wild-type
KO: Knockout
MFI: Mean fluorescence intensity
MZ: Marginal zone
pBtk: Phosphorylated Bruton tyrosine kinase
pCD19: Phosphorylated CD19
pSHIP: Phosphorylated SH2-containing inositol phosphatase
pWASP: Phosphorylated Wiskott–Aldrich syndrome protein
F-actin: Filamentous actin
sAg: Soluble antigen
TIRFm: Total internal reflection fluorescent microscopy
WASP: Wiskott–Aldrich syndrome protein
