# Supplementary figures and images for: AKT2 deficiency impairs formation of the BCR signalosome
Source: Cell Commun Signal. 2020 Apr 6;18:56. doi: 10.1186/s12964-020-00534-9 (PMC7133013; doi:10.1186/s12964-020-00534-9)

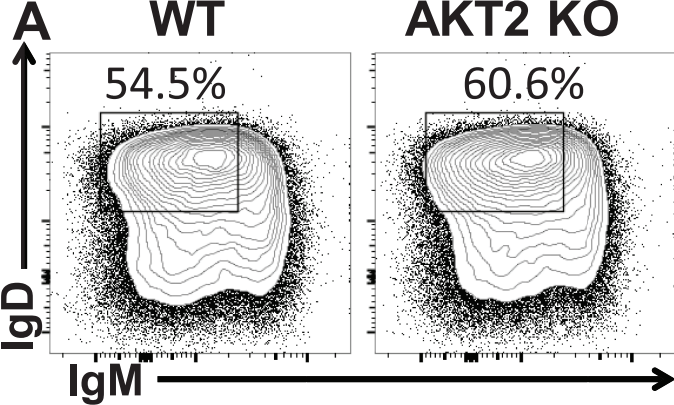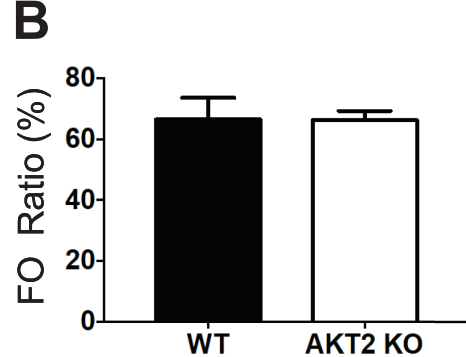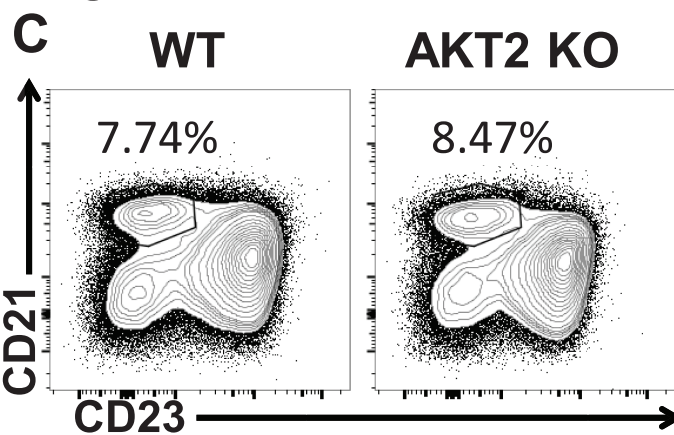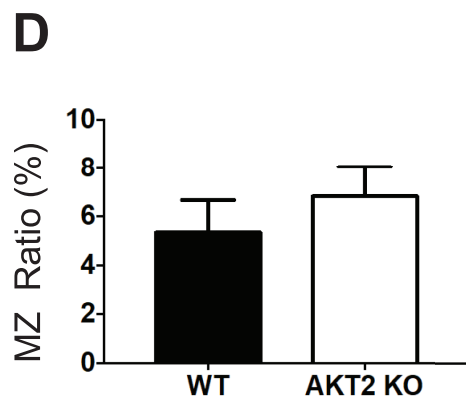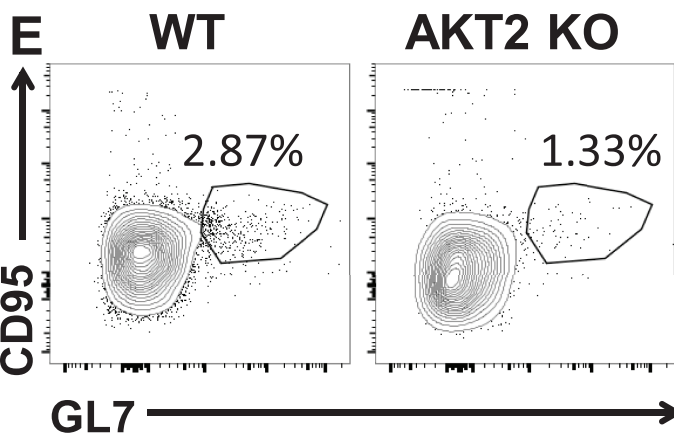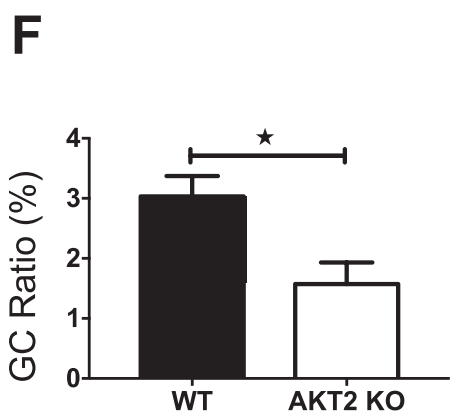

Supplement: Supplementary file 2 — Additional file 1: Fig. S1. The effect of AKT2 deficiency on the germinal center (GC) B cells is cell-intrinsic. CD45.1 mice were irradiated with sublethal radiation (6Gy), and then intravenously injected with 1 × 10^7^ total BM cells containing AKT2 deficient BM (CD45.2) and WT BM (CD45.1) at a ratio of 1:1. After 8 weeks, B cells from the chimera mice were labeled with specific antibodies for surface marker of FO (A), MZ (C) and GC (E) B cells, and analyzed using flow cytometry (n = 3). Shown are the frequency (+SEM) of different populations (B, D, F). T-test was used to do the statistics, *p < 0.01. Fig. S2. The gene expression of Btk, Was and Cd19 is comparable in splenic B cells from WT and AKT2 KO mice. The total RNA was isolated from the splenic B cells from WT and AKT KO mice (n = 3). The gene expression of Btk (A), Was (B) and Cd19 (C) were detected by real-time PCR, and Gapdh was used to standardized the mRNA expression. T-test was used to do the statistics. [file 12964_2020_534_MOESM1_ESM.zip › Supplementary Figure S1.pdf]

**A**

relative mRNA level

*Btk*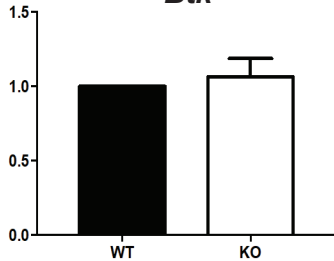**B**

relative mRNA level

*Was*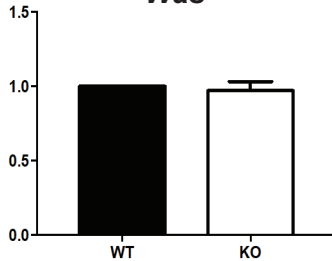**C**

relative mRNA level

*Cd19*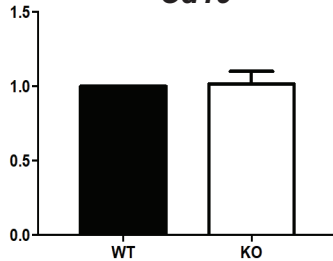

Supplement: Supplementary file 2 — Additional file 1: Fig. S1. The effect of AKT2 deficiency on the germinal center (GC) B cells is cell-intrinsic. CD45.1 mice were irradiated with sublethal radiation (6Gy), and then intravenously injected with 1 × 10^7^ total BM cells containing AKT2 deficient BM (CD45.2) and WT BM (CD45.1) at a ratio of 1:1. After 8 weeks, B cells from the chimera mice were labeled with specific antibodies for surface marker of FO (A), MZ (C) and GC (E) B cells, and analyzed using flow cytometry (n = 3). Shown are the frequency (+SEM) of different populations (B, D, F). T-test was used to do the statistics, *p < 0.01. Fig. S2. The gene expression of Btk, Was and Cd19 is comparable in splenic B cells from WT and AKT2 KO mice. The total RNA was isolated from the splenic B cells from WT and AKT KO mice (n = 3). The gene expression of Btk (A), Was (B) and Cd19 (C) were detected by real-time PCR, and Gapdh was used to standardized the mRNA expression. T-test was used to do the statistics. [file 12964_2020_534_MOESM1_ESM.zip › Supplementary Figure S2.pdf]
